# Concomitant parasite infections influence tuberculosis immunopathology and favor rapid sputum conversion of pulmonary tuberculosis patients

**DOI:** 10.1186/s12941-024-00756-6

**Published:** 2024-11-07

**Authors:** Augustine Yeboah, Monikamira Vivekanandan, Ernest Adankwah, Dorcas O. Owusu, Wilfred Aniagyei, Difery Minadzi, Isaac Acheampong, Joseph F. Arthur, Millicent Lamptey, Mohammed K. Abass, Francis Kumbel, Francis Osei-Yeboah, Amidu Gawusu, Linda Batsa Debrah, Alexander Debrah, Ertan Mayatepek, Julia Seyfarth, Richard O. Phillips, Marc Jacobsen

**Affiliations:** 1 Kumasi Centre for Collaborative Research in Tropical Medicine (KCCR), Kumasi, Ghana; 2 Agogo Presbyterian Hospital, Agogo, Ghana; 3 St Mathias Catholic Hospital, Yeji, Ghana; 4 Atebubu District Hospital, Atebubu, Ghana; 5 Sene West Health Directorate, Kwame Danso, Ghana; 6 Department of General Pediatrics, Neonatology and Pediatric Cardiology, University Hospital Duesseldorf, Medical Faculty, Heinrich-Heine-University Duesseldorf, Moorenstr. 5, 40225 Duesseldorf, Germany; 7 School of Medicine and Dentistry, College of Health Sciences, Kwame Nkrumah University of Science and Technology (KNUST), Kumasi, Ghana

**Keywords:** Tuberculosis, Co-infections, IL-6, Treatment efficacy

## Abstract

Immunopathology of human tuberculosis (TB) in a subgroup of patients is characterized by aberrantly high concentrations of inflammatory cytokines, for example Interleukin (IL)-6. Concomitant (co-)infections by parasites can affect host immunity, but the impact on immunopathology in TB patients is poorly defined. Here we characterized a group of patients with TB ( n = 76) from Ghana with different protozoan and helminth co-infections. Plasma cytokines were measured at the onset of disease and anti-mycobacterial treatment efficacy was monitored during disease course. A subgroup of TB patients had co-infections with protozoan (n = 19) or helminth (n = 16) parasites. Plasma analyses for candidate cytokines identified lower levels of IL-6 in parasite co-infected patients with TB. Moreover, it took less time for co-infected patients to become sputum-negative for *Mycobacterium tuberculosis* during treatment. These results indicated an influence of parasite co-infections on immunopathology in TB and suggested positive effects on treatment efficacy.

## Introduction

*Mycobacterium tuberculosis*, the causative agents for tuberculosis (TB), as well as various parasites (e.g., protozoa, helminth) of humans are frequently found in sub-Saharan Africa. In this region parasite co-infections are inevitable, and these have been shown to affect TB disease manifestation and severity in previous studies [1]. There is ample evidence that interference between immune responses against different types of infectious agents contributes to this phenomenon [2]. Immune polarization and regulation are important mechanisms, which have been studied in detail [3, 4].

In contrast less is known about possible effects of parasite co-infections on immunopathology found in some chronic infectious diseases like COVID19. Here, immunopathology is characterized by aberrant strong cytokine secretion with harmful side effects [5–8]. Especially, patients with severe COVID19 are prone to develop immunopathology with often deadly outcomes [8]. Host-directed treatment regimens have been shown to be helpful in patients with severe COVID19 and immunosuppressive drugs as well as biologicals against key cytokines, like IL-6, showed promising results [9–11]. Immunopathology is also seen in a subgroup of patients with pulmonary TB and these show hyperinflammation accompanied by impaired immune responses in the blood [5, 12–14]. Disease severity and treatment outcome have been found to be associated with immunopathology in patients with TB [15–17]. Moreover, a study by DiNardo et al. identified a gene signature of blood immunopathology in different cohorts of TB patients and provided evidence that immunopathology classifies distinct endotypes of pulmonary TB in humans [18]. Key cytokines of inflammation are increased in the TB immunopathology, with IL-6 emerging as a particularly promising marker [19–22]. As a result of the inflammatory cytokine milieu in the blood, constitutive phosphorylation of STAT3 and high concentrations of the key inhibitor SOCS3 were found to impair the T cell response in TB patients [13, 23]. High IL-6 plasma concentrations at disease onset were associated with impaired T cell responses and delayed sputum conversion in TB patients [21, 22, 24].

In the present study, we recruited and characterized a study group of patients with TB for the occurrence of several parasite co-infections, including protozoan and helminth pathogens. The diverse picture of co-infections prompted us to determine effects in all parasite co-infected individuals as well as in subgroups of either protozoan or helminth co-infected individuals compared to patients with TB without detected co-infections. Plasma cytokine levels and sputum conversion during anti-mycobacterial treatment were compared between the cohorts.

## Methods

### Recruitment and diagnosis of patients with TB

We recruited patients with pulmonary TB (n = 76) between April 2019 and September 2021 at the Agogo Presbyterian Hospital, the St Mathias Catholic Hospital, the Atebubu District Hospital, and the Sene West District Hospital in Ghana. Diagnosis of acute TB was based on patient history, clinical examination, chest X-ray, as well as sputum tests for acid-fast bacilli. All patients had chest X-ray and clinical symptoms suggestive of TB. Laboratory tests (i.e., sputum smear, culture, GeneXpert) confirmed diagnosis for all TB patients. All patients were sputum positive for *M. tuberculosis* at baseline, and this was the first known episode of TB for all included patients. Heparinized blood as well as sputum samples were taken prior to initiation of treatment. In addition, sputum samples were retrieved at different time points after onset of treatment (i.e., 6, 9, 12, and 16 weeks) and were analyzed by smear microscopy and culture. Not all patients were present at the different time points and contamination of mycobacterial culture (Becton Dickinson BACTEC™ MGIT™, USA) also affected the number of sputum results (i.e., mycobacteria positive or negative) per time point. The numbers of included sputum tests are indicated in Fig. 2a.

### Diagnosis of parasite co-infection

All included patients were comprehensively analyzed for co-infections by different parasite species. Whole blood tests for rapid detection of antibodies against *Plasmodium falciparum* (ALERE™ Malaria Pf HRP2 Ag), *Onchocerca volvulus* and *Wuchereria bancrofti* (Standard Diagnostics BIOline^TM^ Oncho/LF IgG4 biplex test kit) were performed according to manufacturers’ instructions to screen for *P. falciparum*, *O. volvulus* and *W. bancrofti* infections***.*** In addition, blood microscope analyses for identification of *O. volvulus, W. bancrofti* and *Mansonella perstans* microfilariae was done. In brief, whole blood (1000 µl) was passed through a Whatman filter and the membrane was fixed with methanol (3 ml). Thereafter the filter membrane was washed with distilled water and dried on a slide overnight. Giemsa staining was then performed and the slides were examined under a light microscope at 10 to 40 × magnification.

Stool and urine examinations were performed for detection of intestinal and urinary parasites (i.e., *Giardia lamblia, Ascaris lumbricoides*, *hookworms* (*Ancylostoma duodenale/Necator americanus*), *Strongyloides stercoralis*, *Schistosoma mansoni*). In brief, stool (1 g) was fixed in 10% formalin (10 ml) and the suspension was filtered through a sieve into a falcon tube. Then diethyl ether (3 ml) was added and centrifuged at 300 g for 5 min. The supernatant was discarded and two smears were taken on glass slides, which were then examined under the microscope at 10 to 40 × magnification for ova, cysts or larvae of intestinal parasites. Urine (10 ml) was centrifuged at 300 g for 5 min and the supernatant was discarded thereafter. A remaining drop of sediment was then re-suspended and pipetted onto slide covered with a coverslip. The sediment slide was examined under the microscope at 10 to 40 × magnification for ova, cysts or larvae of intestinal parasites.

On this basis TB patients without co-infections (n = 41) or with any parasite co-infection (n = 35) were classified. TB patients with parasite co-infections were further subdivided into protozoa-infected (n = 19) and helminth-infected (n = 16) subgroups. Co-infections with more than one protozoa/helminth species were not seen. Patient subgroup characteristics are summarized in Table 1.

**Table 1:** Study group characteristics

	TB patients (w/o co-infection)	TB patients (with co-infection)		
		Parasite (i.e., Protozoa, Helminths)	Protozoa	Helminths
Total number, n	41	35	19	16
Age, median (range)	54 (15–89)	41 (14–80)	44 (28–80)	35.5 (14–80)
Males, n (%)	26 (63.4)	22 (62.8)	15 (78.9)	7 (43.8)*
Symptoms				
Cough > 2 wks, n (%)	38 (92.7)	34 (97.1)	19 (100)	15 (93.8)
Fever, n (%)	10 (24.4)	7 (20.0)	5 (26.3)	2 (12.5)
Chest pain, n (%)	14 (34.1)	9 (25.7)	5 (26.3)	4 (25.0)
Hemoptysis, n (%)	9 (21.1)	5 (14.3)	3 (15.8)	2 (12.5)
Weight loss, n (%)	8 (19.5)	10 (28.6)	7 (36.8)	3 (18.8)

*Sex differences between protozoa and helminth co-infected TB patients were significant; Fisher’s exact test, p = 0.0043. n, number; w/o, without

### Plasma cytokine analysis using the cytometric bead assay (CBA)

Blood plasma was enriched from heparinized blood (5ml) from each study participant as described before [22]. The LEGENDplex™ Multi-Analyte Flow Assay kit (Custom Human Assay, BioLegend, USA) was used for the simultaneous detection of cytokines (i.e., IL-6, IP-10, IL-22, IL-10, GM-CSF, IFNγ, IL-8) in plasma samples according to manufacturer’s instructions and as previously described [22]. Samples were measured using a CytoFlex S flow cytometer (Beckman Coulter, USA) and data were analyzed using the cloud version of the Biolegend LEGENDplex Data Analysis Software (Qognit. Inc). Values below the standard curve were set to 1pg/ml for depiction and calculations.

### Statistical tests

All statistical analyses were performed using GraphPad Prism v9 software (GraphPad Software, La Jolla CA, USA). Against the background of moderate study group sizes, we performed non-parametric tests throughout. The Mann–Whitney U-test was applied for plasma cytokine comparisons between the study groups. For comparison of sputum positive and sputum negative proportions at individual time points the Fisher exact test was performed. For comparison of median time frames until sputum conversion, the Logrank (Mantel-Cox) test was done. A p-value below 0.05 was considered statistically significant. Graphs were generated using GraphPad Prism version 9.

## Results and discussion

A total number of 76 patients with confirmed pulmonary TB recruited at the Ashanti region in Ghana were analyzed for endemic parasite co-infections with different helminth and protozoan species. Altogether 35 patients with TB (46.1%) had parasite co-infections and nine pathogens (seven helminth and two protozoan species) were found. *P. falciparum* was by far the most frequent co-infection (n = 15; 42.9%) and together with *G. lamblia* (n = 4; 11.4%), these two protozoan species accounted for the majority of parasite co-infections in this cohort.

Helminth co-infections revealed a heterogeneous picture and several species were detected with low frequency. Only *A. lumbricoides* was detected in more than 10% (n = 4; 11.4%) of parasite co-infected patients. The other helminths were hookworms (n = 3; 8.6%), *O. volvulus* (n = 2; 5.8%), *W. bancrofti* (n = 2; 5.8%), *S. stercoralis* (n = 2; 5.8%), *M. perstans* (n = 2; 5.8%), and *S. mansoni* (n = 1; 2.9%). Given the low frequency of most pathogen species, we decided to compare the study group of TB patients without detected co-infection with those co-infected with any parasite (i.e., protozoa or helminth) or with subgroups co-infected with a parasite from the protozoan or helminth group. Characteristics of these subgroups are shown in Table 1.

Sex distributions were comparable between TB patients with- and without parasite co-infection but a lower proportion of males was seen in the helminth co-infected group, when compared to protozoa co-infected patients (p = 0.043). Next, we compared clinical symptoms of TB between the study groups; no significant differences were seen. The results suggested no significant impact of co-infections on disease severity in TB patients.

**Fig. 1:**
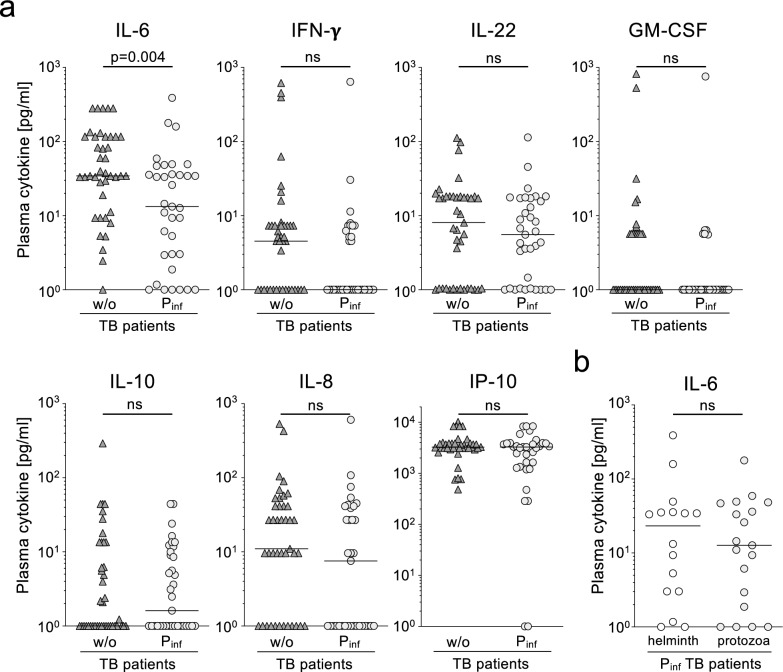
Plasma cytokine comparisons between TB patients with and without parasite infections.** a** Blood plasma levels of seven cytokines (i.e., IL-6, IFN-γ, IL-22, GM-CSF, IL-10, IL-8, IP-10) measured by cytometric beads array (CBA) were compared in TB patients with parasites (P_inf_; bright grey circles, n = 41) or without (w/o) parasite co-infections (grey triangles, n = 35). **b** Subgroups of parasite co-infected TB patients with detected helminth (n = 16) and protozoa species (n = 19) were compared for IL-6 plasma concentrations. **a**, **b** Symbols indicate mean values of duplicates measured for individual patients. Study group median values are depicted as lines. Nominal p-values of the Mann-Whitnes U-test are given for significant differences (p < 0.05)

Previous studies identified cytokine patterns in the plasma of TB patients that reflected immunopathology of affected pulmonary tissue [12]. Hence, we measured the expression of seven plasma cytokines and compared this between the study groups. Six cytokines (i.e., IP-10, IL-8, IL-22, IFN-g, IL-10, GM-CSF) had similar median concentrations between the study groups of parasite co-infected and non-co-infected patients (Fig. 1a). However, significantly lower IL-6 concentrations were detected in the plasma of patients with TB which were co-infected with parasites (Fig. 1a).

**Fig. 2:**
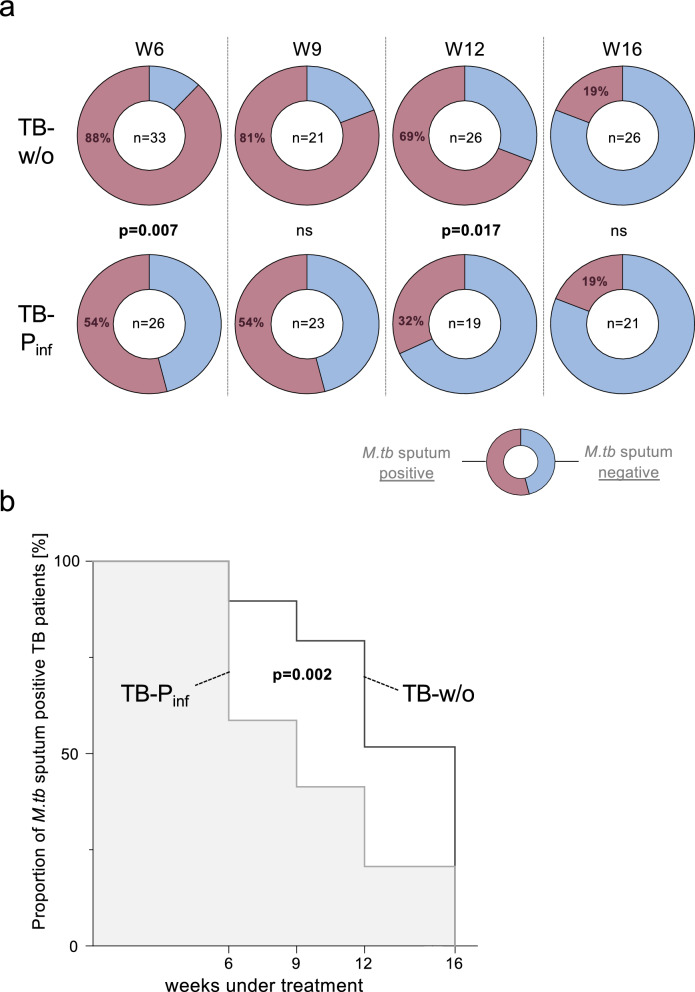
Sputum conversion analyses for TB patients with and without parasite infections. **a** Sputum analyses (smear and culture) for mycobacteria was performed at different time points after onset of treatment (i.e., 6, 9, 12, 16 weeks). Median proportions of positive (red color) and negative (blue color) tests as well as the number (n) of analyzed samples are given as pie charts for both subgroups of TB patients with parasite (TB-P_inf_) and without parasite (TB-w/o) co-infections. Nominal p-values of the Fisher exact test are given for significant differences (p < 0.05). **b** Time courses of sputum test results are depicted as Kaplan-Meyer survival curves. Durations until median sputum negativity for* M. tuberculosis* were compared between the study groups with parasite co-infection (grey line, bright grey background color) and without parasite co-infection (black line, open background) using Logrank test. A nominal p-value is given. ns: not significant

To characterize the influence of different parasite co-infections, we next compared the cytokines between protozoa and helminth co-infected subgroups. As a result, no differences were seen between the subgroups for IL-6 (Fig. 1b) or the other cytokines (data not shown). IL-6 has been described to be a key marker of immunopathology in the plasma of patients with TB, with initial studies providing evidence that IL-6 can be used to monitor treatment efficacy in tuberculosis patients [22, 24]. Hence, we next determined sputum samples from all individuals for detection of mycobacteria prior to treatment and at different time points after onset of anti-mycobacterial treatment. All patients had positive sputum samples prior to treatment onset but already after six weeks parasite co-infected individuals showed significantly lower proportions of positive tests (54%) as compared to non-parasite co-infected individuals (88%) (p = 0.007; Fig. 2a). Lower proportions of positive sputum tests were also seen at later time points although only significant for week 12 (p = 0.017; Fig. 2a). Against this background, we determined the time frames until sputum conversion for individual patients and compared durations between the study groups. Co-infected tuberculosis patients showed a more rapid response to anti-mycobacterial treatment as compared to individuals without parasite co-infections (p = 0.002; Fig. 2b). A 50% median reduction was reached between week six and nine for parasite co-infected TB patients whereas non-parasite co-infected patients reached 50% median reduction at week 16 (Fig. 2b). Similar differences are seen for the subgroups of helminth and protozoa co-infected subgroups (data not shown). These results were in accordance with previous studies that showed more rapid treatment response in tuberculosis patients with low IL-6 plasma levels [22].

Our data suggests that co-infections with parasites affect immunopathology seen in a subgroup of patients with TB. Immunopathology can be interpreted as a consequence of immune hypersensitivity to a particular infection and *M. tuberculosis* infection is the prototype of delayed-type hypersensitivity, characterized by dominant cellular immune activation and inflammation. Therefore, we hypothesize that helminth and/or protozoan co-infection reduces the effects of hypersensitivity-induced harmful immunopathology seen in patients with acute pulmonary TB [5, 12]. Parasite co-infections had no significant effect on the severity of the disease but shortened the time frame to sputum conversion during anti-mycobacterial treatment. These results are of clinical importance since the shortening of treatment requirements is an important goal in efforts to eradicate human TB.

## Data Availability

Data is provided within the manuscript.

## References

[1] Babu S, Nutman TB. Helminth-tuberculosis co-infection: an immunologic perspective. Trends Immunol. 2016;37(9):597–607. 27501916 10.1016/j.it.2016.07.005 PMC5003706

[2] Mabbott NA. The influence of parasite infections on host immunity to co-infection with other pathogens. Front Immunol. 2018;9:2579. 30467504 10.3389/fimmu.2018.02579 PMC6237250

[3] Page KR, Scott AL, Manabe YC. The expanding realm of heterologous immunity: friend or foe? Cell Microbiol. 2006;8(2):185–96. 16441430 10.1111/j.1462-5822.2005.00653.x

[4] Supali T, Verweij JJ, Wiria AE, Djuardi Y, Hamid F, Kaisar MM, Wammes LJ, van Lieshout L, Luty AJ, Sartono E, Yazdanbakhsh M. Polyparasitism and its impact on the immune system. Int J Parasitol. 2010;40(10):1171–6. 20580905 10.1016/j.ijpara.2010.05.003

[5] Amaral EP, Vinhaes CL, Oliveira-de-Souza D, Nogueira B, Akrami KM, Andrade BB. The interplay between systemic inflammation, oxidative stress, and tissue remodeling in tuberculosis. Antioxid Redox Signal. 2021;34(6):471–85. 32559410 10.1089/ars.2020.8124 PMC8020551

[6] Diorio C, Henrickson SE, Vella LA, McNerney KO, Chase J, Burudpakdee C, Lee JH, Jasen C, Balamuth F, Barrett DM, Banwell BL, Bernt KM, Blatz AM, Chiotos K, Fisher BT, Fitzgerald JC, Gerber JS, Gollomp K, Gray C, Grupp SA, Harris RM, Kilbaugh TJ, John ARO, Lambert M, Liebling EJ, Paessler ME, Petrosa W, Phillips C, Reilly AF, Romberg ND, Seif A, Sesok-Pizzini DA, Sullivan KE, Vardaro J, Behrens EM, Teachey DT, Bassiri H. Multisystem inflammatory syndrome in children and COVID-19 are distinct presentations of SARS-CoV-2. J Clin Invest. 2020;130(11):5967–75. 32730233 10.1172/JCI140970 PMC7598044

[7] Mortaz E, Tabarsi P, Varahram M, Folkerts G, Adcock IM. The immune response and immunopathology of COVID-19. Front Immunol. 2020;11:2037. 32983152 10.3389/fimmu.2020.02037 PMC7479965

[8] Que Y, Hu C, Wan K, Hu P, Wang R, Luo J, Li T, Ping R, Hu Q, Sun Y, Wu X, Tu L, Du Y, Chang C, Xu G. Cytokine release syndrome in COVID-19: a major mechanism of morbidity and mortality. Int Rev Immunol 2021:1–14 10.1080/08830185.2021.1884248 PMC7919105 33616462

[9] Maeurer M, Ramalho R, Wang FS, Zumla A. Host-directed therapies for COVID-19. Curr Opin Pulm Med. 2021;27(3):205–9. 33629969 10.1097/MCP.0000000000000769

[10] Wallis RS, O’Garra A, Sher A, Wack A. Host-directed immunotherapy of viral and bacterial infections: past, present and future. Nat Rev Immunol. 2023;23(2):121–33. 35672482 10.1038/s41577-022-00734-z PMC9171745

[11] Liu B, Li M, Zhou Z, Guan X, Xiang Y. Can we use interleukin-6 (IL-6) blockade for coronavirus disease 2019 (COVID-19)-induced cytokine release syndrome (CRS)? J Autoimmun. 2020;111: 102452. 32291137 10.1016/j.jaut.2020.102452 PMC7151347

[12] Ahor HS, Vivekanandan M, Harelimana JD, Owusu DO, Adankwah E, Seyfarth J, Phillips R, Jacobsen M. Immunopathology in human pulmonary tuberculosis: Inflammatory changes in the plasma milieu and impaired host immune cell functions. Immunology. 2024. 10.1111/imm.13761. 38317426 10.1111/imm.13761

[13] Adankwah E, Seyfarth J, Phillips R, Jacobsen M. Aberrant cytokine milieu and signaling affect immune cell phenotypes and functions in tuberculosis pathology: What can we learn from this phenomenon for application to inflammatory syndromes? Cell Mol Immunol. 2021;18(8):2062–4. 34035497 10.1038/s41423-021-00695-8 PMC8144869

[14] Ellner JJ. Immunosuppression in tuberculosis. Infect Agents Dis. 1996;5(2):62–72. 8721043

[15] Kumar NP, Moideen K, Nancy A, Viswanathan V, Thiruvengadam K, Nair D, Banurekha VV, Sivakumar S, Hissar S, Kornfeld H, Babu S. Plasma chemokines are baseline predictors of unfavorable treatment outcomes in pulmonary tuberculosis. Clin Infect Dis. 2021;73(9):e3419–27. 32766812 10.1093/cid/ciaa1104 PMC8563183

[16] Kumar NP, Moideen K, Nancy A, Viswanathan V, Thiruvengadam K, Sivakumar S, Hissar S, Nair D, Banurekha VV, Kornfeld H, Babu S. Association of plasma matrix metalloproteinase and tissue inhibitors of matrix metalloproteinase levels with adverse treatment outcomes among patients with pulmonary tuberculosis. JAMA Netw Open. 2020;3(12): e2027754. 33258908 10.1001/jamanetworkopen.2020.27754 PMC7709089

[17] Kumar NP, Velayutham B, Nair D, Babu S. Angiopoietins as biomarkers of disease severity and bacterial burden in pulmonary tuberculosis. Int J Tuberc Lung Dis. 2017;21(1):93–9. 28157471 10.5588/ijtld.16.0565 PMC6340056

[18] DiNardo AR, Gandhi T, Heyckendorf J, Grimm SL, Rajapakshe K, Nishiguchi T, Reimann M, Kirchner HL, Kahari J, Dlamini Q, Lange C, Goldmann T, Marwitz S, et al. Gene expression signatures identify biologically and clinically distinct tuberculosis endotypes. Eur Respir J. 2022;60(3):2102263. 35169026 10.1183/13993003.02263-2021 PMC9474892

[19] Oh JY, Lee YS, Min KH, Hur GY, Lee SY, Kang KH, Rhee CK, Park SJ, Shim JJ. Elevated interleukin-6 and bronchiectasis as risk factors for acute exacerbation in patients with tuberculosis-destroyed lung with airflow limitation. J Thorac Dis. 2018;10(9):5246–53. 30416771 10.21037/jtd.2018.08.29 PMC6196218

[20] Losada PX, Perdomo-Celis F, Castro M, Salcedo C, Salcedo A, DeLaura I, Lastra G, Narvaez CF. Locally-secreted interleukin-6 is related with radiological severity in smear-negative pulmonary tuberculosis. Cytokine. 2020;127: 154950. 31864093 10.1016/j.cyto.2019.154950

[21] Gupte AN, Kumar P, Araujo-Pereira M, Kulkarni V, Paradkar M, Pradhan N, Menon P, Padmapriyadarsini C, Hanna LE, Yogendra Shivakumar SVB, Rockwood N, Du Bruyn E, Karyakarte R, Gaikwad S, Bollinger R, Golub J, Gupte N, Viswanathan V, Wilkinson RJ, Mave V, Babu S, Kornfeld H, Andrade BB, Gupta A. Baseline IL-6 is a biomarker for unfavourable tuberculosis treatment outcomes: a multisite discovery and validation study. Eur Respir J. 2022;59(4):2100905. 34711538 10.1183/13993003.00905-2021 PMC7612881

[22] Vivekanandan MM, Adankwah E, Aniagyei W, Acheampong I, Yeboah A, Arthur JF, Lamptey MNK, Abass MK, Gawusu A, Kumbel F, Osei-Yeboah F, Debrah LB, Owusu DO, Debrah A, Mayatepek E, Seyfarth J, Phillips RO, Jacobsen M. Plasma cytokine levels characterize disease pathogenesis and treatment response in tuberculosis patients. Infection. 2023;51(1):169–79. 35759173 10.1007/s15010-022-01870-3 PMC9879809

[23] Harling K, Adankwah E, Guler A, Afum-Adjei Awuah A, Adu-Amoah L, Mayatepek E, Owusu-Dabo E, Nausch N, Jacobsen M. Constitutive STAT3 phosphorylation and IL-6/IL-10 co-expression are associated with impaired T-cell function in tuberculosis patients. Cell Mol Immunol. 2019;16(3):275–87. 30886421 10.1038/cmi.2018.5 PMC6460487

[24] Vivekanandan MM, Adankwah E, Aniagyei W, Acheampong I, Minadzi D, Yeboah A, Arthur JF, Lamptey M, Abass MK, Kumbel F, Osei-Yeboah F, Gawusu A, Debrah LB, Owusu DO, Debrah A, Mayatepek E, Seyfarth J, Phillips RO, Jacobsen M. Impaired T-cell response to phytohemagglutinin (PHA) in tuberculosis patients is associated with high IL-6 plasma levels and normalizes early during anti-mycobacterial treatment. Infection. 2023;51(4):1013–23. 36650358 10.1007/s15010-023-01977-1 PMC10352402

